# A customized anthropomorphic 3D-printed phantom to reproducibility assessment in computed tomography: an oncological case study

**DOI:** 10.3389/fonc.2023.1123796

**Published:** 2023-08-28

**Authors:** Carlo Cavaliere, Dario Baldi, Valentina Brancato, Marco Aiello, Marco Salvatore

**Affiliations:** IRCCS Synlab SDN, Naples, Italy

**Keywords:** 3D printing, computed tomography, phantom, lung tumor, polylactic acid

## Abstract

**Introduction:**

Studies on computed tomography (CT) reproducibility at different acquisition parameters have to take into account radiation dose administered and related ethical issues. 3D-printed phantoms provide the possibility to investigate these features deeply and to foster CT research, also taking advantage by outperforming new generation scanners. The aim of this study is to propose a new anthropomorphic 3D-printed phantom for chest lesions, tailored on a real patient CT scan, to investigate the variability of volume and Hounsfield Unit (HU) measurements at different CT acquisition parameters.

**Methods:**

The chest CT of a 75-year-old patient with a paramediastinal lung lesion was segmented based on an eight-compartment approach related to HU ranges (air lung, lung interstitium, fat, muscle, vascular, skin, bone, and lesion). From each mask produced, the 3D.stl model was exported and linked to a different printing infill value, based on a preliminary test and HU ratios derived from the patient scan. Fused deposition modeling (FDM) technology printing was chosen with filament materials in polylactic acid (PLA). Phantom was acquired at 50 mAs and three different tube voltages of 80, 100, and 120 kVp on two different scanners, namely, Siemens Somatom Force (Siemens Healthineers, Erlangen, Germany; same setting of real patient for 80 kVp acquisition) and GE 750 HD CT (GE Healthcare, Chicago, IL). The same segmentation workflow was then applied on each phantom acquisition after coregistration pipeline, and Dice Similarity Coefficient (DSC) and HU averages were extracted and compared for each compartment.

**Results:**

DSC comparison among real patient *versus* phantom scans at different kVp, and on both CT scanners, demonstrated a good overlap of different compartments and lesion vascularization with a higher similarity for lung and lesion masks for each setting (about 0.9 and 0.8, respectively). Although mean HU was not comparable with real data, due to the PLA material, the proportion of intensity values for each compartment remains respected.

**Discussion:**

The proposed approach demonstrated the reliability of 3D-printed technology for personalized approaches in CT research, opening to the application of the same workflow to other oncological fields.

## 1 Introduction

Phantoms are frequently employed to offer a real-world test environment for evaluating and ensuring the quality of medical imaging equipment (1, 2). Commercial phantoms frequently contain materials with realistic tissue Hounsfield Unit (HU) values, but they frequently have straightforward, generic shapes and sizes that do not closely reflect real patients, making it challenging to compare the imaging system’s performance between phantoms and humans. Nevertheless, the usefulness of 3D-printed phantoms in radiodiagnostic and interventional research has been proven (3–5). In experiments using ionizing radiation, they are especially useful in experiments with repeated exposures ethically acceptable for humans. In addition, phantoms enable comprehensive portrayal of complicated anatomies and combined with different post-processing and visualization techniques (e.g., cinematic rendering and AI-driven algorithms) are filling the gap between technologically complex state-of-art scanners and clinical specialists, supporting diagnostic, preoperative, and educational purposes (6, 7).

Many attempts have been performed to employ the 3D printing of lung and thorax for reliable phantoms (2, 8–12). Jung et al. (2) constructed lung sub-volumes with a cylindrical form, printed using polylactic acid (PLA; density 1.25 g/cm^3^), tailored to fit inside a commercial phantom. In this case, ribs and spinal structures were not included in the phantom. Kairn et al. (8) employed acrylonitrile butadiene styrene (ABS; density 1.05 g/cm3) to print a lung phantom, but excluding vertebrae, ribs, and airways from the printing model. Mayer et al. (9) used a multi-materials (tango plus and vero white for soft tissue and bone, respectively) jetting additive manufacturing printer to create a thorax phantom, excluding lung tissue, blood veins, or airways from the model, and introducing a mobile tumor in the design. Pallotta et al. (10), designed a phantom employing an ABS-printed body, with simplified internal organ forms, and calcium sulfate dehydrate-filled ribs, with the lungs represented by two cork foil blocks and lung tumors represented by a moldable bolus. Using white nylon, Larsson et al. created a premature infant’s lung phantom, with empty internal structures to fill with a liquid phantom (11). Finally, Hazelaar et al. (12), starting from a clinical CT scan, created an assembled multi-material (gypsum, nylon, and silicone) life-size phantom with three lung tumors poured into a 3D-printed mold. The latter work, although with an anatomy more detailed, proposes a multi-material approach that makes the printing deployment complex and expensive, both in terms of the 3D printing system and the cost and availability of different materials. Overall, actual 3D phantom solutions are commonly not modeled starting from a real CT dataset, and/or pathological CT. Moreover, they reproduce only parts of the thorax (in the majority of cases exclusively the lungs, limited structures such as air and lung parenchyma, or a thick slice) (13–16). In addition, they often represent a black box in terms of reversing engineering processes and infill/architecture percentages, avoiding the reproducibility of 3D-printed phantom production and assessment.

The aim of this study was to create a low-cost patient-tailored chest phantom, strictly representing multi-tissue anatomy of a real patient with oncological lesion. Based on an eight-compartment approach, we evaluate the spatial accuracy and HU values differences at different CT acquisition parameters and scanners; they have been evaluated on a 3D-printed prototype of the thoracic district.

## 2 Methods

**Figure 1:**
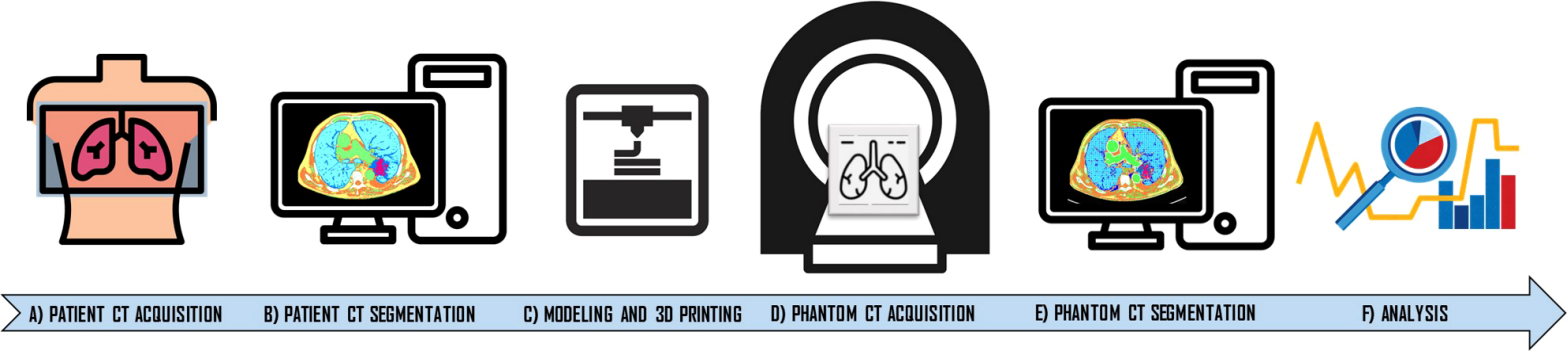
Workflow of the manufacturing approach developed in the study. The approach starts with the chest computed tomography (CT) acquisition of the patient **(A)**. CT patient images were segmented using an eight-compartment approach related to HU ranges (air lung, lung interstitium, fat, muscle, vascular, skin, bone, and lesion) **(B)**. Then, phantom modeling and 3D printing was performed **(C)**. Phantom was acquired **(D)** and segmented and the same segmentation workflow was applied **(E)**. Dice Similarity Coefficient (DSC) and HU averages were extracted and compared for each compartment **(F)**.

The overall methodological workflow of the proposed approach is shown in **Figure 1**.

### 2.1 Real imaging dataset for 3D modeling

From the chest CT acquisition of a 75-year-old patient with a bioptic diagnosis of non-small cell lung cancer, we selected the post-contrast portal venous phase in order to maximize the tissue contrast and mainly lesion heterogeneities.

The CT was acquired with a Siemens Somatom Force scanner (Siemens Healthineers, Erlangen, Germany) at 50 mA, 80 kVp, with a 3-mm slice thickness and a resolution of 0.6 mm × 0.6 mm. There was a lung tumor in the inferior half of the left lung, the maximum axial diameter was ~6.2 cm, and the neighbor pleura was involved.

### 2.2 Phantom 3D model and 3D printing

CT real dataset was imported to a dedicated segmentation software (inPrint, Materialise NV, Leuven). Eight masks were defined according to HU ranges, using the threshold and/or region growing tools. In details, air lungs range from −1024 to −749 HU, lung interstitium from −750 to −125 HU, fat from −124 to −25 HU, muscle from −24 to 150 HU, vascular tree from 70 to 225 HU, and bone and calcifications from 226 to 2400 HU. Moreover, in order to increase segmentation accuracy, a dedicated mask was defined for the lung lesion, subdividing a more vascularized part along the inferior-medial border (range 61–374 HU), from a less post-contrast enhanced lesion region (range from −425 to −375 HU).

**Figure 2:**
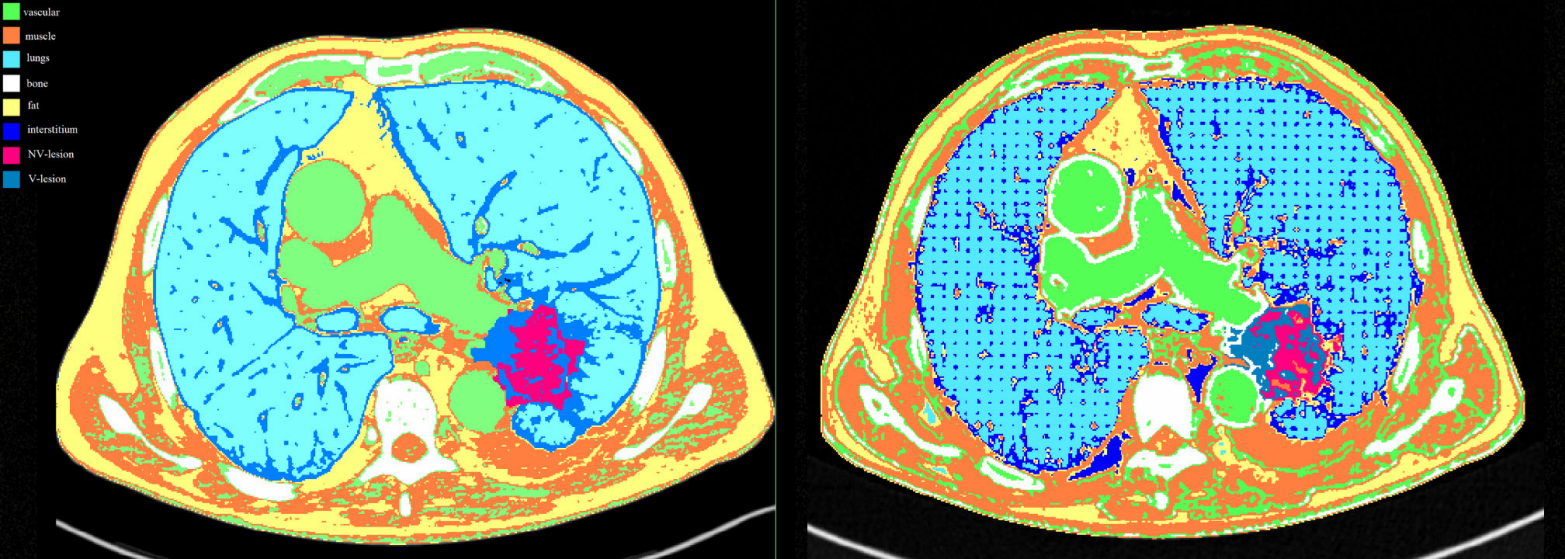
Masks comparison for each compartment between the patient (left image) and the phantom (right image) acquired at 80 kVp on Siemens scanner. Color legend was green for the vascular component, orange for the muscles, light blue for the lungs, white for the bones, yellow for the fat, blue for the interstitium, fuchsia for the NV-lesion, and royal blue for the V-lesion.

The masks covered the entire HU range of the voxels, so that no gaps were left empty during 3D printing (**Figures 2**).

**Figure 3:**
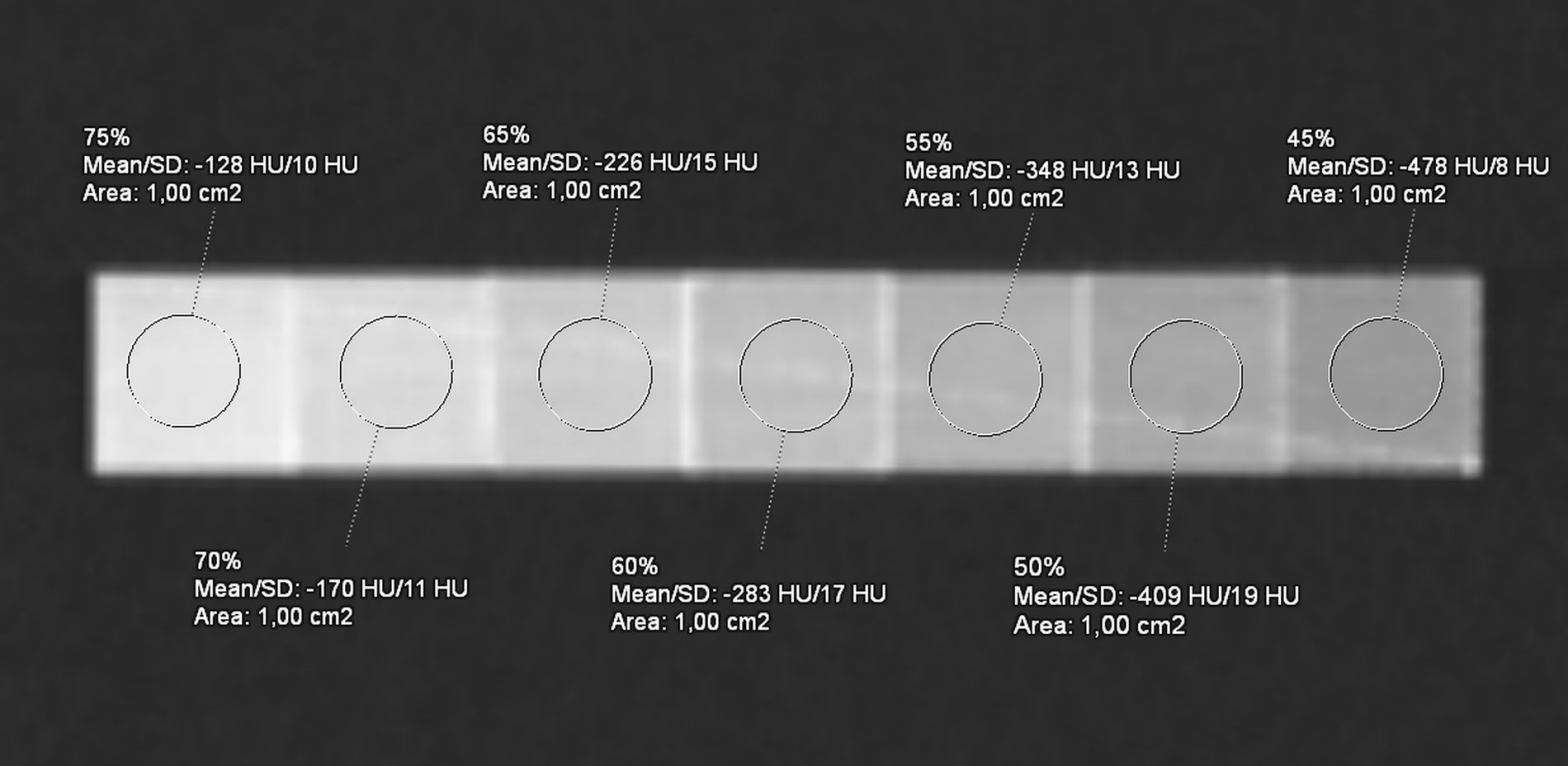
Computed tomography (CT) acquisition of the reference phantom with seven distinct PLA infill percentage levels, ranging from 75% to 45%, with a step of 5%. The average HU is reported by the ROI (1 cm^2^) on each cube (2 cm side).

From each mask produced, the 3D STL (Standard Triangulation Language) model was exported to the open-source CURA 5.2.1 software (Ultimaker, Netherlands) for slicing. A previous 3D printing test was performed to relate each 3D model (air lung, lung interstitium, fat, muscle, vascular, skin, bone, and lesion) with an infill value. Briefly, we associated at the two extremes, air and bone, respectively, the values of 0% and 100% PLA infill and evaluated HU measures derived by different infill rates with a range of 5% (**Figure 3**). Following, according to HU averages extracted by each compartment of the real dataset, we proportionally chose the infill rates of the phantom masks, selecting 10% for “air lungs,” 35% interstitium, 40% fat, 55% muscle, 70% vascular, and 50% and 62.5% for the two components of the lung lesion, respectively, for not-vascularized (NV-Lesion) and vascularized (V-Lesion) components.

The value for lungs was set up at 10% and not 0% to guarantee a minimal scaffold for the support of other phantom components without significantly affecting HU measures (**Figure 2**).

Subsequently, the STL file was converted into layers (generating contour data), producing the toolpaths on XYZ axes to fill them and determining the filament’s quantity to be used in the manufacturing of the product. The toolpath was defined by G-code files generated for each layer of the product, and these codes provided the instruction for the X, Y, and Z motions of the tool to generate the required layers. All STL preprocessing steps for fused deposition modeling (FDM) technology printing were performed on the CURA software, including machine platform, heating of the printing plate to promote adhesion, adjusting orientation, repairing parts, generating necessary supports, and positioning the model.

**Figure 4:**
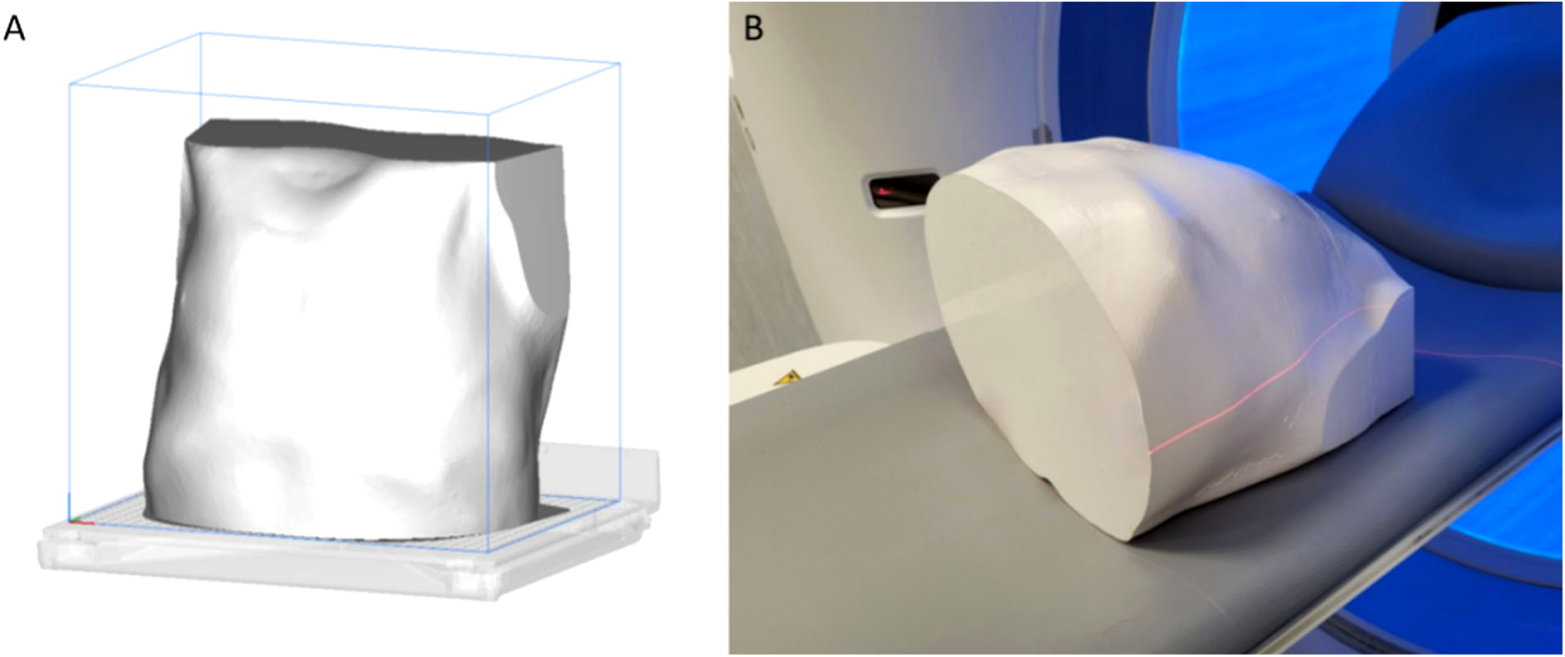
In **(A)**, the representation of the whole phantom on the 3D-printing plate, after the slicing. In **(B)**, the phantom on the scanner bed before the Siemens acquisition.

The filament material chosen was PLA, preferred by most domestic 3D printers worldwide, because it is easy to print for its extruding temperature ranging between 180°C and 210°C (17). The PLA material chosen for the 3D printing has a cost of about €30 for kilograms (final phantom weight of about 9 kg; printing times: about 3 days). The 3D printing of the samples was performed using a desktop 3D printer, Ultimaker 5S (Ultimaker, Netherlands) (**Figure 4**). The use of more expensive industrial 3D printers could fast the whole process.

The model, once printed, is acquired at 50 mAs and three different tube voltages of 80, 100, and 120 kVp on two different scanners, namely, Siemens Somatom Force (Siemens Healthineers, Erlangen, Germany) and GE 750 HD CT (GE Healthcare, Chicago, IL) with mediastinal window.

**Figure 5:**
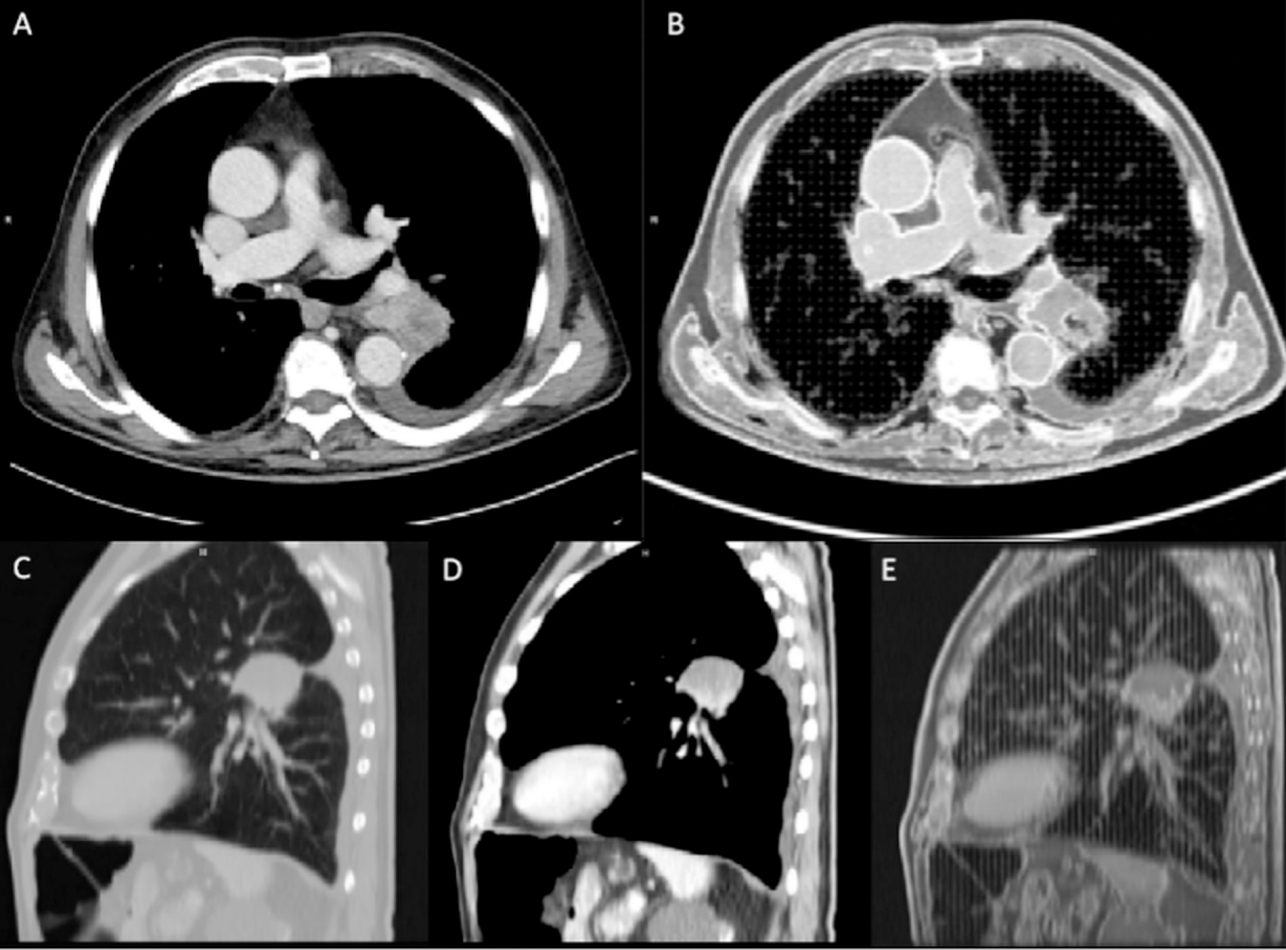
Computed tomography (CT) comparison between the patient **(A, C, D)** and the phantom **(B, E)** on an axial and sagittal plane. The two components (vascularized and non-vascularized lesion) were clearly depicted in the sagittal plane **(D, E)**. Images were acquired on the Siemens scanner at 80 kVp.

On these acquisitions, segmentation was again performed, with the same previous software and workflow, starting from the acquisition performed on Siemens Force of the 80 kVp phantom, achieved with the same acquisition parameters and scanner of the patient (**Figure 5**). The HU ranges were then used for the segmentation of the remaining structures, encompassing for air lungs (−1024/−809 HU), lung interstitium (−808/−684), fat (−808/−615), muscle (−614/−357), vascular (−356/−237), bone and calcifications (−236/−361), and two components of lesion (V-Lesion 586/−432, and NV-Lesion −431/−237). The lung interstitium mask, partially overlapped with fat HU ranges, and was segmented with the threshold value function and differentiated through the region growing tool by the fat mask. Similarly, two submasks were defined for the lesion to differentiate the V-Lesion, partially overlapping with the vascular and muscle component, and the NV-Lesion, partially overlapping with the muscle. Boolean subtraction operations were finally performed to subtract partially overlapping masks.

### 2.3 Image processing

The CT images of the phantom were co-registered in the space of the original CT (which coincides with the space of the 3D printing plane) by means of a rigid registration procedure performed by using Elastix software (v. 4.9.0, http://elastix.isi.uu.nl/). A four-level multiresolution approach using a Gaussian smoothing without downsampling was applied. A localized version of mutual information was considered as the similarity measure, consisting in evaluating mutual information on multiple subregions. Specifically, the localization is obtained by constraining the sampling procedure to a cubic subregion of the image, randomly chosen in every iteration step from the fixed image domain (18, 19). The standard gradient descent was applied for metric optimization (20). The calculated transformation following the co-registration was used to transform other images and the corresponding masks using the same transformation parameters by means of the Transformix tool (21).

### 2.4 Reproducibility evaluation

The results of the segmentations were compared against the segmentations of the print bed to evaluate variability and accuracy for each tissue compartment. The Dice Similarity Coefficient (DSC) was used as an evaluation metric of the spatial overlap between the compartment-based segmentations. Furthermore, the variability of the signal in terms of mean and standard deviation was evaluated for each segmentation.

## 3 Results

**Table 1** shows the results concerning the segmentation accuracy. The top average DSC scores achieved were those obtained for lung masks (DSC = 0.9 on average), regardless of the acquisition scanner and tube voltage. High values of DSC were also obtained for the lesion, both vascularized and non-vascularized (0.66 < DSC < 0.82). DSC values obtained for fat, muscle, bone, and vascular masks were all between 0.56 and 0.58. The lowest values of DSC were obtained for interstitium (0.37 < DSC < 0.48).

**Table 1:** Dice Similarity Coefficient results between patient masks and phantom masks obtained performing the acquisition with two different scanners and using tube voltages of 80, 100, and 120 kVp.

	SIEMENS			GE		
	80 kVp	100 kVp	120 kVp	80 kVp	100 kVp	120 kVp
**fat**	0.56	0.55	0.56	0.57	0.57	0.57
**interstitium**	0.38	0.37	0.48	0.44	0.44	0.43
**V-lesion**	0.8	0.79	0.82	0.82	0.66	0.81
**NV-lesion**	0.73	0.73	0.73	0.73	0.77	0.69
**muscle**	0.57	0.57	0.56	0.57	0.56	0.56
**bone**	0.58	0.57	0.56	0.58	0.58	0.58
**lungs**	0.9	0.89	0.9	0.9	0.89	0.89
**vascular**	0.56	0.56	0.56	0.56	0.56	0.55

**Table 2**; **Figure 6** showed compartment-based values and distributions of HU values, both for patient and phantom CT images. HU recorded on phantom showed reduced values, mainly negative, compared with the real dataset, with a reduced variability within the compartment. The closest HUs values were extracted for the lung (−868 and about −925 for real and phantom acquisition, respectively) and for the interstitium (−518 and about −723 for real and phantom acquisition, respectively). No significant differences were found for HUs values between scanners and among different parameter settings.

**Table 2:** Mean and standard deviation of signal intensities values (given in HU) in CT images of patient and phantom (acquired on two different scanners, using tube voltages of 80, 100, and 120 kVp).

	PATIENT	PHANTOM					
		SIEMENS			GE		
	80 kVp	80 kVp	100 kVp	120 kVp	80 kVp	100 kVp	120 kVp
**Fat**	−82.02 ± 28.87	−658.75 ± 56.72	−657.77 ± 55.87	−656.75 ± 55.46	−643.17 ± 113.21	−642.79 ± 106.38	−643.41 ± 101
**Interstitium**	−518.86 ± 191.52	−727.52 ± 67.85	−726.23 ± 67.48	−724.37 ± 66.69	−725.37 ± 75.17	−719.7 ± 75.4	−718.37 ± 75.83
**V-lesion**	84.4 ± 28.55	−361.11 ± 58.63	−359.87 ± 59.15	−356.65 ± 56.12	−358.18 ± 63.01	−355.08 ± 60.24	−353.47 ± 61.05
**NV-lesion**	−36.27 ± 142.68	−506.53 ± 55.44	−508.97 ± 55.31	−503.22 ± 52.05	−505.96 ± 73.14	−502.99 ± 69.43	−503.26 ± 71.31
**Muscle**	29.54 ± 26.34	−474.81 ± 80.13	−471.14 ± 80.55	−469.16 ± 79.16	−473.61 ± 96.74	−469 ± 94.9	−468.31 ± 94.9
**Bone**	470.78 ± 199.89	−147.23 ± 96.69	−136.81 ± 101.34	−132.16 ± 103.66	−184.17 ± 145.21	−176.17 ± 145.63	−172.53 ± 147.35
**Lungs**	−868.57 ± 44.76	−932.64 ± 63.12	−933.78 ± 64.03	−933.17 ± 63.89	−919.68 ± 58.57	−919.71 ± 60.31	−921.15 ± 61.11
**Vascular**	138.72 ± 42.75	−304.1 ± 47.22	−298.12 ± 45.99	−294.87 ± 43.93	−308.82 ± 68.49	−302.6 ± 64.75	−299.42 ± 65.08

**Figure 6:**
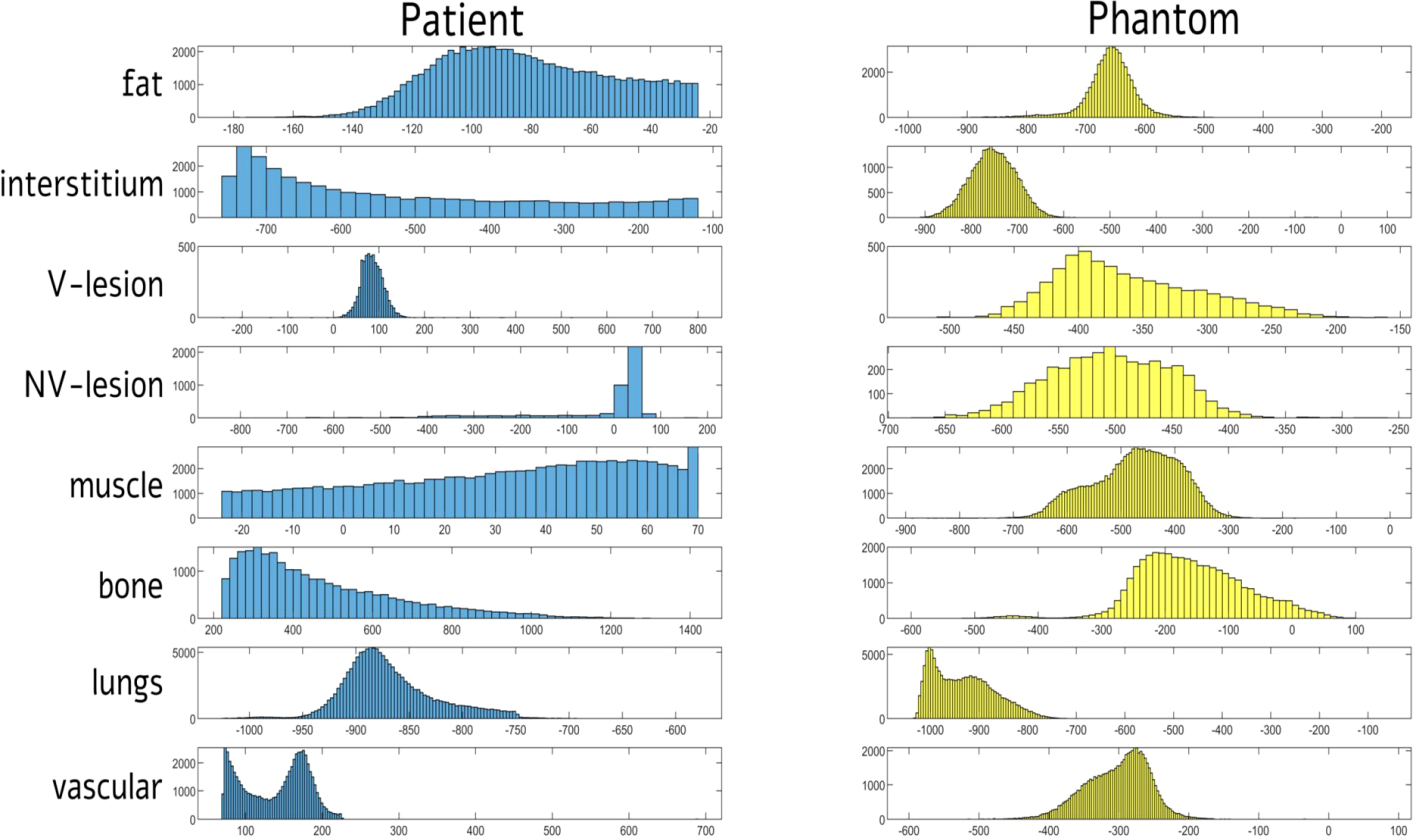
Distribution of signal intensities (HU) for each of eight tissue compartments in computed tomography (CT) images of patient and phantom acquired on Siemens scanner at 80 kVp. HUs = Hounsfield Units; V = vascularized; NV = non-vascularized.

**Figure 7:**
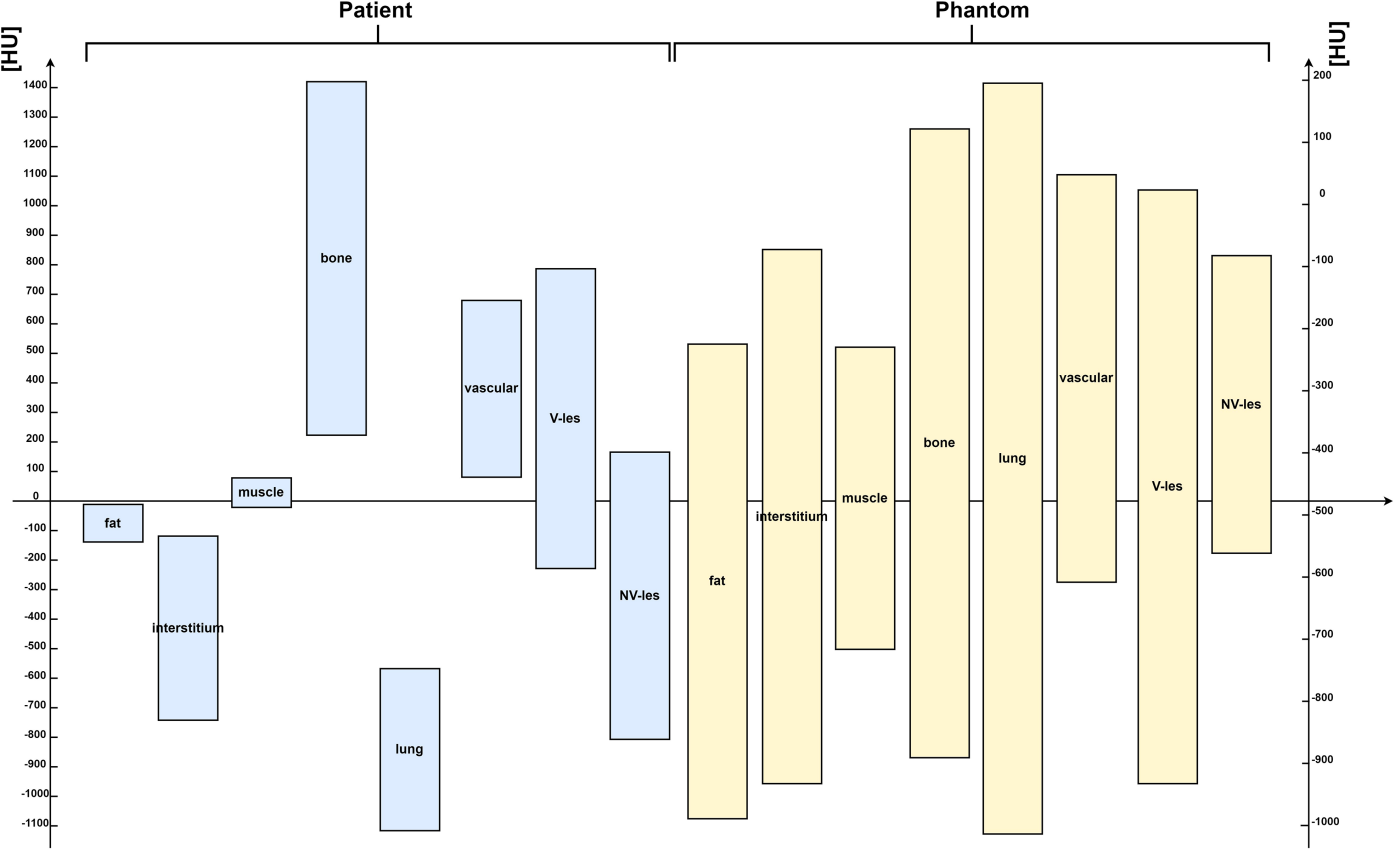
Hounsfield scale diagram of computed tomography (CT) values for the eight investigated compartments, both for patient (on the left side) and for phantom CT images (on the right side) acquired on Siemens scanner at 80 kVp. HUs = Hounsfield Units.

**Figure 7** showed the comparison of Hounsfield scale diagram of CT values for the eight investigated compartments between patient and phantom CT images. As shown in the latter figure, the examined compartment tissues on the phantom had a Hounsfield scale trend similar to the patient.

## 4 Discussion

In this work, we propose a simple additive manufacturing approach for the definition of anthropomorphic chest phantoms to be used for the study of reproducibility in CT.

The approach presented constitutes a rapid, economical, and customizable solution to mimic different structures and tissues in a CT environment, thus supporting the experimentation and validation of different CT image acquisition and processing approaches.

Together with a technical description that guides the design and printing of the 3D model, the work proceeds with an effective characterization of the phantom representative of the thoracic district through reproducibility measurements at variable acquisition parameters (kVp) and manufacturers.

The results show an excellent agreement among the measurements obtained, both in terms of volume and in terms of signal. As regards the accuracy of the delineation of the tissues with respect to the reference of the printed model, the results estimated through the Dice coefficient, although highly reproducible, remain on average low. Compared with the literature presented in the introduction, only one other study made a comparison between the segmentation of the printing plate and that of the phantom result, obtaining a Dice coefficient of 0.87 for the target region of cardiac left ventricle (22), comparable with the result obtained for the lung region, but not for the others, following our approach. This result may be mainly due to the inevitable constructive limitations of the phantom; in fact, the higher the number of different fabrics to be printed, the greater the overlap between the relative printing HUs. Furthermore, a well-known limitation of deposition printing is the need to create a scaffold to enable printing in the presence of empty areas, such as in the lung regions, where, as mentioned in the introduction, it is impossible to achieve −1000 HUs. The infill rate of 10% chosen in our approach affects both DSC and HU measures, reducing volumetric overlapping of lungs for the introduction of the “unreal” scaffold and increasing the interstitium mask. Moreover, this supports, although minimal, affected also HU measures increasing values of both the structures. This kind of scaffold was applied also by Jung et al. (2), which used PLA (density 1.25 g/cm³) to print lung tissue and the volume that is presumably air inside the lung filling with 0.3-mm strips as a supporting structure. Similarly, quantitative parameters and mainly the DSC values were conditioned by the interface between different masks, as evident for the phantom vascular one, where the superimposition of neighbor compartments determined an artificial higher values outline that the automatic HU-related segmentation assigned to bone mask. Unfortunately, the choice of materials with HUs more “real” is biased by the lack of systematic evidences investigating HUs correlates of different materials, according also to changing percentage of infill, obliging toward more expensive and professional multi-materials printers.

Indeed, the main limitation of the study is the absence of equivalence in terms of HU between real tissues and phantom tissues, although similar HU ratios were maintained, even if almost all negative for the phantom and excluding lung and interstitium for the biases mentioned before.

Unfortunately, the difference between the reference HU values and the ones achieved with the phantom represents a limit of actual desktop 3D printer technologies and not of the defined approach, describing an easy and cheap workflow starting from a real CT dataset until to the creation of a patient-tailored whole thorax phantom.

A better equivalence could be obtained by using different materials for each tissue (12), including more electron-dense ones, but it is actually not achievable with common desktop 3D printers and cheaper consumables. Indeed, it would involve a significant increase in costs, violating the cost-effectiveness requirement of the proposed approach, needed to build patients’ tailored models. A first further step would be a systematic assessment of CT HU values of the available materials, in order to better set up the workflow [e.g., more recent filaments with mixed metallic additives (23)].

Conversely, the high DSC detected for the lesion sub-compartments suggests the possibilities of this model for oncological studies, highlighting 3D printing heterogeneity accuracy for smaller masks, useful for examples for radiomics studies reproducibility and repeatability (24, 25). In particular, given the ability of radiomic in noninvasively capturing tumor heterogeneity that is essential for grading, treatment planning, and following-up oncological patients, this study pave the way for future studies looking for repeatable radiomic features (e.g., texture, wavelet-transformed, Laplacian of Gaussian-filtered) and involving phantoms simulating heterogeneities in different imaging modalities (26, 27).

Moreover, the proposed manufacturing approach can also be extended to a variety of other districts and other imaging modalities such as MRI and PET (28–31).

Considering the importance of dose reduction in CT examinations, the proposed approach deserves further characterization to evaluate its use for optimizing the dose delivered during CT protocols, as done in other studies more focused on this aspect (3, 32–34). To achieve this, experiments could be planned in which both current (mA) and voltage (kVp) parameters and, at the same time, the impact on the signal-to-noise ratio of the obtained images are controlled.

Unlike other studies in which lung phantoms only included limited structures such as air and lung parenchyma, or made assessments on a single slice print (13–16), the developed phantom comprises all the structures that make up the thoracic region. In particular, the implemented comprehensive representation includes not only the lung but also surrounding structures, thus providing a more realistic simulation of the entire chest anatomy.

However, it should be considered that creating a 3D-printed phantom of the entire thoracic region presents significant challenges compared with printing a single thick slice. In particular, printing such a large and complex structure requires considerable time, resources, and expertise. On the other hand, the complexity of our phantom design allowed us to accurately simulate the behavior of radiation when interacting with the printed volume, making it more suitable for dosimetric evaluations in CT acquisitions (33,34).

At the same time, it should be considered that the PLA material employed in our study is one of the most popular materials used in 3D printing due to its safety, affordability, ease of printing, and outstanding material properties. The use of PLA for printing the phantom is therefore cost-effective, offering the advantage of utilizing common and economical materials without compromising on the quality of the printed object.

## 5 Conclusion

In conclusion, the proposed approach provides a simple and cost-effectiveness procedure for 3D printing with conventional fused deposition modelling, starting from any CT exam, useful for reproducibility oncological studies in the CT environment. The automated workflow could be applied to compare/optimize different CT acquisition protocols on the phantom and to evaluate the impact/reproducibility of different quantitative CT algorithms (e.g., radiomics features) as the CT acquisition protocol changes, which is not feasible on a real patient. This approach can be extended to other anatomical regions and imaging modalities, and future research should focus on dose optimization and the improvement of tissue delineation accuracy. Overall, our work contributes a practical and effective tool for advancing medical imaging and oncology research.

## Data availability statement

The raw data supporting the conclusions of this article will be made available by the authors, without undue reservation.

## Ethics statement

The study was conducted according to the guidelines of the Declaration of Helsinki, and approved by the Ethics Committee of the Istituto Nazionale Tumori “Fondazione G. Pascale (protocol number 7/20).

## Author contributions

CC designed the study, drafted and revised the manuscript. DB created the model from the CT, printed the phantom and performed the segmentations. VB performed the analyses and drafted the manuscript. MA drafted and revised the manuscript. MS supervised the manuscript and provided administrative support. All authors have read and agreed to the published version of the manuscript.
